# Brain activation upon ideal-body media exposure and peer feedback in late adolescent girls

**DOI:** 10.3758/s13415-017-0507-y

**Published:** 2017-05-04

**Authors:** Mara van der Meulen, Jolanda Veldhuis, Barbara R. Braams, Sabine Peters, Elly A. Konijn, Eveline A. Crone

**Affiliations:** 1 Department of Psychology, Faculty of Social Sciences, Leiden University, Wassenaarseweg 52, 2333 AK Leiden, The Netherlands; 2 Leiden Institute for Brain and Cognition, Leiden, The Netherlands; 3 Department of Communication Science, Media Psychology Program, VU University Amsterdam, Amsterdam, The Netherlands

**Keywords:** Peer influence, Media effects, Ideal-body imagery, Adolescents, Body image, FMRI, Self-esteem

## Abstract

Media’s prevailing thin-body ideal plays a vital role in adolescent girls’ body image development, but the co-occurring impact of peer feedback is understudied. The present study used functional magnetic resonance imaging (fMRI) to test media imagery and peer feedback combinations on neural activity related to thin-body ideals. Twenty-four healthy female late adolescents rated precategorized body sizes of bikini models (*too thin* or *normal*), directly followed by ostensible peer feedback (*too thin* or *normal*). Consistent with prior studies on social feedback processing, results showed increased brain activity in the dorsal medial prefrontal cortex (dmPFC)/anterior cingulate cortex (ACC) and bilateral insula in incongruent situations: when participants rated media models’ body size as *normal* while peer feedback indicated the models as *too thin* (or vice versa). This effect was stronger for girls with lower self-esteem. A subsequent behavioral study (*N* = 34 female late adolescents, separate sample) demonstrated that participants changed behavior in the direction of the peer feedback: precategorized *normal* sized models were rated as *too thin* more often after receiving *too thin* peer feedback. This suggests that the neural responses upon peer feedback may influence subsequent choice. Our results show that media-by-peer interactions have pronounced effects on girls’ body ideals.

Adolescence, which is the age period between approximately 10 and 22 years, is an important developmental period for social reorientation and identity development (Steinberg, 2008). Media content matters in social development, especially for adolescents who socialize largely with their peers in media(ted) environments (e.g., Facebook; Brown & Bobkowski, 2011; Konijn, Veldhuis, Plaisier, Spekman, & Den Hamer, 2015). This is well exemplified by the development of body image in late adolescent girls. Thin-ideal body portrayals are overrepresented in contemporary media fare (e.g., Grabe, Ward, & Hyde, 2008; Lopez-Guimera, Levine, Sanchez-Carracedo, & Fauquet, 2010), while the prevalence of overweight and obesity is still increasing (WHO, 2015). This discrepancy underscores how bodies as they appear in media are not only unrealistic (especially in comparison to the actual female population; Fouts & Burggraf, 2000) but also unattainable (as graphic software is heavily used to adapt body shapes to be ultra-slender and toned; Derenne & Beresin, 2006). Late adolescent girls form a particularly sensitive group to internalize the thin-body ideal (Grabe et al., 2008; Groesz, Levine, & Murnen, 2002; Veldhuis, Konijn, & Seidell, 2012), and may subsequently experience negative body affects, such as body dissatisfaction. Indeed, it has been shown that body dissatisfaction increases across adolescence, reaching its highest level in late adolescence (Bearman, Presnell, Martinez, & Stice, 2006; Bucchianeri, Arikian, Hannan, Eisenberg, & Neumark-Sztainer, 2013).

Among late adolescent girls, not only media but also peers are important intensifiers of body-ideal perceptions (Jones & Smolak, 2011; Keery, van den Berg, & Thompson, 2004). Research shows that peer feedback is likely vital in shaping adolescent girls’ ideas about body ideals. For example, peer comments indicating thin-ideal media models to be “only a few kilos underweight” resulted in negative body perceptions among adolescent girls (Veldhuis, Konijn, & Seidell, 2014b). Concurrently, studies in the field of developmental neuroscience have demonstrated the importance of peer feedback in relation to social norms (e.g., Crone, Will, Overgaauw, & Güroğlu, 2014). How media exposure and peer feedback interact in influencing late adolescents’ body standards is not well understood, but neural measures might be a useful tool in investigating the underlying mechanisms of this relationship, as it is less sensitive to socially desirable answers. The present study examined brain activation upon exposure to media model imagery followed by peer feedback.

The neural correlates of peer feedback have previously been studied using social judgment paradigms (Gunther Moor, van Leijenhorst, Rombouts, Crone, & Van der Molen, 2010). In these studies, participants were presented a picture of a peer who had evaluated them based on their online profile, and they were subsequently asked to indicate whether they thought the peer liked them based on a first impression. This judgment was followed by ostensible peer feedback, which could be congruent or incongruent with the participant’s answer (i.e., “I expect to be accepted,” followed by peer feedback signaling acceptance [congruent] or rejection [incongruent]). These studies revealed common activity in the anterior cingulate cortex (ACC) and insula when receiving incongruent feedback (Gunther Moor et al., 2010; Guyer et al., 2014; Somerville, Heatherton, & Kelley, 2006). This ACC-insula network is also typically observed when individuals make social choices that are different from their own social norm (Guroglu, van den Bos, Rombouts, & Crone, 2010; van den Bos, van Dijk, Westenberg, Rombouts, & Crone, 2011). Additionally, prior studies in adults demonstrated that the ACC-insula is more active when participants receive incongruent feedback from peers about music popularity (Berns, Capra, Moore, & Noussair, 2010), music preference (Campbell-Meiklejohn, Bach, Roepstorff, Dolan, & Frith, 2010) and attractiveness of faces (Klucharev, Hytonen, Rijpkema, Smidts, & Fernandez, 2009), suggesting that activity in the ACC-insula network reflects deviance of (social) norms (Rilling & Sanfey, 2011) or deviance from (social) expectations (Somerville et al., 2006). Thus, the current *personal perception* versus *peer feedback* paradigm provides a promising method to examine how late adolescent girls process peer feedback on ideal-body media imagery that is either consistent or inconsistent with their own personal judgment, especially when neural responses are measured during such a paradigm. Neural measures can complement self-report measures in predicting behavior (Berkman & Falk, 2013) and reveal mechanisms that are not apparent otherwise (see also Falk, 2010).

We performed two experiments to test these hypotheses in two samples of female late adolescents. The first study (fMRI study) tested systematic variations in neural activity (ACC and insula) following (perceived) body sizes of the portrayed media models (i.e., being too thin or normal) and subsequent peer feedback (i.e., too thin or normal). We tested if *incongruent* situations in which the peer feedback did *not* resemble the girls’ own perception of the media model (i.e., too thin vs. normal or normal vs. too thin) compared to *congruent* situations (i.e., too thin vs. too thin and normal vs. normal) would lead to increased activity in the ACC and insula. Prior research in adults confirmed that participants changed their behavior following peer feedback on face attractiveness, suggesting that the neural signaling in response to peer feedback may influence subsequent choice preference (Klucharev et al., 2009; Zaki, Schirmer, & Mitchell, 2011). Therefore, our second study (behavioral study), in a different late adolescent female sample, tested if participants changed their ratings of the models after receiving peer feedback.

A third goal was to test for the role of self-esteem. Previous studies have indicated that adolescents with lower self-esteem appear to be more at risk for body image concerns, which seems related to less positive body perceptions (Ferguson, 2013; Roberts & Good, 2010). In contrast, adolescent girls with lower self-esteem reported less negative body affect after being informed about the underweight status of ultrathin models (Veldhuis, Konijn, & Seidell, 2014a). These findings imply that girls with lower self-esteem are not only more sensitive for body image concerns but are also more responsive toward feedback on media portrayals. Therefore, we expected to find stronger neural activity for incongruent feedback in participants with lower self-reported self-esteem, as well as a larger change in behavior following peer feedback. Finally, we performed an exploratory analysis to the role of body dissatisfaction in the influence of peer feedback on rating media models.

## Experiment 1

### Method

#### Participants

The sample for the fMRI study consisted of 24 healthy female participants ages 18 to 19 years (*M*_age_ = 19.1 years, *SD*_age_ = .5 years). The sample size was based on prior studies using the social judgment paradigm (Somerville et al., 2006). Participants were recruited through local advertisements and through a recruitment website. All participants were screened for MRI contraindications and reported no diagnosed psychiatric disorder using a telephone interview before the scanning session. Informed consent was obtained from participants prior to the scan session. All participants’ height and weight were measured to calculate their BMI (based on weight in kilos divided by the square height in meters; cf. WHO, 2015). This resulted in an average BMI of 22.2 (*SD* = 3.1; range: 17.5–30). Participants received €30 for participation in a larger set of studies. This study was approved by the University’s Medical Ethical Committee and was conducted in accordance with the provisions of the World Medical Association Declaration of Helsinki.

#### Procedure

Before the start of the study, participants were told that they would participate in a study on the processes behind forming judgments. During the lab visit, participants were instructed about the procedure of an fMRI scan. A short explanation about the Body Image Paradigm was provided, followed by six practice trials of the Body Image Paradigm. Participants were weighed and measured before the scanning session. Directly after the scanning session, participants completed a paper-and-pencil version of the questionnaires. Finally, all participants were debriefed by explaining that the feedback provided during the task was not actually based on the opinion of a majority of their peers but merely on the opinion of some of those peers.

#### Task

The Body Image Paradigm is an adapted version of the Social Judgment task, which was previously used by Gunther Moor et al. (2010) and Somerville et al. (2006), in combination with ideal-body imagery format, as previously described by Veldhuis et al. (2012, 2014a). In the paradigm, 60 pretested media models (30 categorized as too thin and 30 as normal; see below) were shown on the scanner screen, and participants rated each model by indicating whether they perceived each of the media models as too thin or as normal. Subsequently, upon their own rating, the participants received feedback indicating opinions from ostensible peers (too thin or normal), as was explained to the participants beforehand. These *own perceptions* versus *peer feedback* combinations of the models’ body sizes led to *incongruent* (too thin–normal and normal–too thin) and *congruent* (too thin–too thin and normal–normal) situations.

##### Precategorization test

In a pretest for the Body Image Paradigm, a large sample of 135 pictures displaying young female media models showing swimwear in beach settings were selected from the Internet (e.g., via Google search images). With Photoshop techniques the images were made comparable in formatting (e.g., cutting areas such that the model was central in the picture and her body image features clearly visible), resulting in a set of similar media model stimuli with a blue sky background, a sandy beach, and the media models facing the camera (cf. Veldhuis et al. (2012, 2014a). An independent sample of young females (*N* = 124; *M* = 15.99; *SD* = 1.90; age range: 12–19) rated each media model on perceived attractiveness and the model’s body size on 10-point semantic scales, varying from *very ugly* to *very beautiful*, and *extremely big* to *extremely thin*. To select stimuli for the Body Image Paradigm, the rated media models were compared on attractiveness and body size (*thin* to *normal weight*). We selected those models that had varying body sizes but were comparable in attractiveness (*M* = 6.55 for models rated as thin, *M* = 6.63 for models rated as normal weight). This resulted in a selection of 30 media models that were rated as too thin and 30 media models that were rated as normal.

##### Experimental task

In the scanner version of the Body Image Paradigm, feedback presented to the participants was randomized, such that participants did not receive the same feedback on the same answer for more than two times in a row. To enhance the credibility of the peer feedback, for 10 models (i.e., 5 times *too thin*, 5 times *normal*) the feedback was fixed to these conditions, because these models were *always* rated as *too thin* or *normal* during the pretest. More specifically, this led to the following set of stimuli: For the 30 *too thin* models, five images were consistently prerated as too thin and therefore always received peer feedback too thin; 25 images were on average prerated as too thin and peer feedback communicated 10 times too thin and 15 times normal. For the 30 normal models, five images were consistently prerated as normal and therefore always received peer feedback normal; 25 images were on average prerated as normal and peer feedback communicated 10 times normal and 15 times too thin. Next, the final conditions included in the analysis were based on the combinations of participants’ own judgments and peer feedback (e.g., participant rating too thin, peer feedback normal), and therefore the number of trials in each condition varied between participants.

**Fig. 1:**
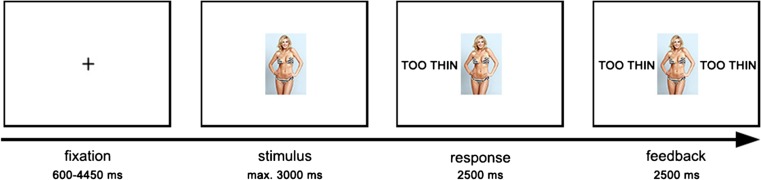
Task sequence of congruent feedback (Color figure online)

During the task, each trial was preceded by a fixation cross with jittered duration between 600 and 4450 ms. Images were presented against a black background for a maximum of 3,000 ms. Within these 3,000 ms, participants had to respond by pressing the left button (too thin) or right button (normal) on a button box with their index or middle finger. Directly after their decision, the choice of the participant (too thin or normal) appeared on the left side of the image on the screen for 2,500 ms. After this, peer feedback (too thin or normal) was presented on the right side of the image for 2,500 ms (see Fig. 1). Responses that exceeded the duration of 3,000 ms were modeled separately and not included in the analyses. Instead, a screen with *Too Slow* was shown 2,500 ms, immediately followed by the start of the next trial. This occurred in less than 3% of the trials.

Several outcome measures were taken from this paradigm. First, we compared the participants’ ratings of the models (*too thin* or *normal*) to our previously set precategorization (*too thin* or *normal*) to check whether their evaluations matched those of our pretest panel. Second, to investigate whether it would take longer to rate models as *too thin* than as *normal*, we calculated the reaction times for these two different response options. Finally, we used the combinations of participants’ ratings and the subsequent peer feedback as conditions in our MRI analyses.

#### Self-report measures

*Self-esteem* was assessed through Rosenberg’s (1965) 10-item Self-Esteem Scale (e.g., “I feel I have a number of good qualities”; as applied in adolescent girls in Veldhuis et al., 2014a), followed by 5-point rating scales (1 = *totally disagree*; 5 = *totally agree*). Higher scores indicated a higher self-esteem. Participants received an average sum score of 26.83 (*SD* = 7.17), with a range of 4–35 (Cronbach’s α = .89).

*Body dissatisfaction* was measured through the 9-item Body Dissatisfaction subscale from the Eating Disorder Inventory (Garner, Olmstead, & Polivy, 1983), which was extended with four items to create a balanced set of indicative (e.g., “I think my belly is too fat”) and counterindicative (e.g., “I am happy with my figure”) answers (cf. Veldhuis et al., 2014a). The 13 items could be answered on a 5-point rating scale (1 = *totally disagree*; 5 = *totally agree*). After recoding, higher scores indicated more body *dis*satisfaction. Participants received a mean score of 32.9 (*SD* = 8.2) with a range of 21–54 (Cronbach’s α = .81).

#### MRI data acquisition

Scans were made with a 3 Tesla Philips scanner, using a standard whole-head coil. The functional scans were acquired using a T2*-weighted echo-planar imaging (EPI). The first two volumes were discarded to allow for equilibration of T1 saturation effects (TR = 2.2 s, TE = 30 ms, sequential acquisition, 38 slices of 2.75 mm, field of view 220 mm, 80 × 80 matrix, in-plane resolution 2.75 mm). After the functional runs, a high resolution 3D T1-weighted anatomical image was collected (TR = 9.751 ms, TE = 4.59 ms, flip angle = 8°, 140 slices, 0.875 mm × 0.875 mm × 1.2 mm, and FOV = 224.000 × 168.000 × 177.333). Visual stimuli were presented on a screen that was attached in the magnet bore. Participants could see the stimuli via a mirror attached to the head coil. Head movement was restricted by using foam inserts inside the coil.

#### FMRI data analysis

All data were analyzed with SPM8 (Wellcome Department of Cognitive Neurology, London). Images were corrected for differences in rigid body motion. Structural and functional volumes were spatially normalized to T1 templates. Translational movement parameters never exceeded 1 voxel (3 mm) in any direction for any participant or scan. The normalization algorithm used a 12-parameter affine transform together with a nonlinear transformation involving cosine basis functions and resampled the volumes to 3 mm cubic voxels. Templates were based on the MNI305 stereotaxic space (Cocosco, Kollokian, Kwan, & Evans, 1997). Functional volumes were spatially smoothed with a 6 mm FWHM isotropic Gaussian kernel.

The onset of the stimulus and the feedback display of each trial were modeled as zero duration events (see Gunther Moor et al., 2010). We divided the feedback displays in four conditions, focusing on combinations of participant ratings with peer feedback: congruent too thin (too thin–too thin), incongruent too thin (too thin–normal), congruent normal (normal–normal) and incongruent normal (normal–too thin).

All events were time locked to the moment of the start of the feedback screen. The trial functions were used as covariates in a general linear model; along with a basic set of cosine functions that high-pass filtered the data. The least-squares parameter estimates of height of the best fitting canonical HRF for each condition were used in pair-wise contrasts. The resulting contrast images, computed on a subject-by-subject basis, were submitted to group analyses. We tested the neural response to incongruent feedback with two contrasts: incongruent thin > congruent thin (and the reversed contrast) and incongruent normal > congruent normal (and the reversed contrast). Task-related responses were considered significant if they exceeded a FWE voxel level threshold of *p* < .05, or a FDR cluster-corrected threshold of *p* < .05, with an initial threshold of *p* < .005 (Woo, Krishnan, & Wager, 2014).

#### Region of interest analysis

We used the MarsBaR toolbox (Brett, Anton, Valabregue, & Poline, 2002) for SPM8 to perform region of interest (ROI) analyses. The general contrast incongruent feedback > congruent feedback, with a threshold of FWE corrected at *p* < .05 at the voxel level, was used to determine suitable ROIs. This contrast was chosen because it is not biased toward *normal* or *too thin* peer feedback, but collapsed across conditions (see Fig. 3). The contrast resulted in three clusters that were extracted with the MarsBaR toolbox: left insula (*x* = −33, *y* = 2-, *z* = −14), right insula (*x* = 36, *y* = 20, *z* = −14) and dorsal medial prefrontal cortex (dmPFC)/ACC (*x* = 0, *y* = 23, *z* = 52). Pearson correlations were computed between the contrast values for dmPFC/ACC, left insula and right insula and self-esteem, BMI and body dissatisfaction.

### Results

#### Behavioral results

First, we tested participants’ ratings of media models as being too thin or normal compared to our initial precategorization of the models as being too thin or normal (i.e., precategorized: too thin vs. participants’ rating: too thin; precategorized: too thin vs. participants’ rating: normal; precategorized: normal vs. participants’ rating: too thin; precategorized: normal vs. participants’ rating: normal). Participants’ ratings of the media models’ body sizes were mostly in accordance with those in the pretest. More specifically, 70.85% of the media models that were precategorized as too thin were also rated as too thin in the main study, while 91.7% of the media models that were precategorized as normal models were rated as normal. It therefore seems that participants were biased towards giving a normal rating to precategorized normal models.

Next, we tested reaction times (RT) for rating the portrayed media models’ body shapes. For this analysis, we only included trials where (1) the model was precategorized as (, 2) the model was precategorized as too thin but participants rated the model as normal, and (3) the model was precategorized as normal and participants rated the model as normal. There were too few trials on which the models were precategorized as normal and participants rated the model as too thin, and therefore these trials were removed from the RT analyses. An ANOVA with the aforementioned three conditions revealed that, regardless of their own ratings of a media model’s body size, it took participants significantly longer to rate the prerated too thin media models’ body sizes (*too thin* model with rating *too thin*: *M* = 1,572 ms, *SD* = 331 ms; *too thin* model with rating *normal*: *M* = 1,626 ms, *SD* = 360 ms) compared to rating precategorized *normal* models (*normal* model with rating *normal*: *M* = 1,304 ms, *SD* = 229), *F*(2, 46) = 17.31, *p* < .001. Thus, even when the participant rated the *too thin* models as *normal,* they were slower to react compared to rating models that were precategorized as *normal* as *normal* (*p* < .001).

#### Body-related measurements

Prior to performing the analyses we checked for outliers in the data. One significant outlier (*Z* value <−3.29 or >3.29; Tabachnick & Fidell, 2013) in self-esteem scores (score 4) was removed from the analyses. This resulted in analyses including 23 participants. There was a significant negative correlation between self-esteem and body dissatisfaction (*r* = −.61, *p* < .005), indicating that a lower self-esteem relates to experiencing more body dissatisfaction. No significant correlations were found between BMI and self-esteem (*r* = −.24, *p* = .26), or BMI and body dissatisfaction (*r* = .36, *p* = .09).

#### Whole brain analysis

**Fig. 2:**
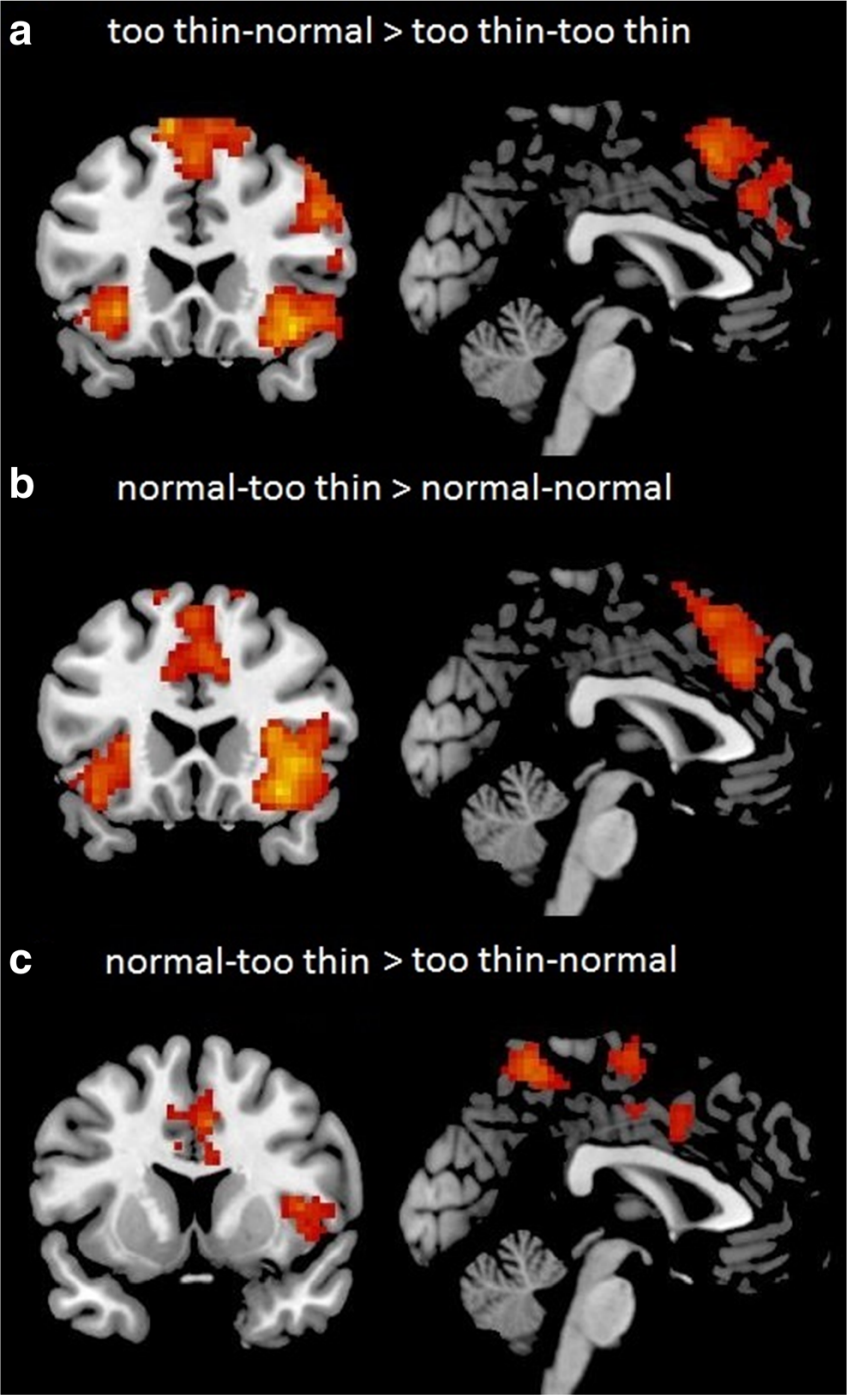
Neural responses in the contrasts (**a**) “participant rating: too thin vs. peer feedback: normal” compared to “participant rating: too thin vs. peer feedback: too thin” and (**b**) in the contrast “participant rating: normal vs. peer feedback: too thin” compared to “participant rating: normal vs. peer feedback: normal,” showing activation in bilateral insula and dmPFC/ACC; (**c**) “participant rating: normal vs. peer feedback: too thin” resulted in stronger activation in the ACC compared to “participant rating: too thin vs. peer feedback: normal” (all contrasts are displayed at FDR cluster-corrected threshold at *p* < .05 with an initial threshold of *p* < .005) (Color figure online)

To test processing of peer feedback that deviated from the participant’s response, we tested neural activation for congruent and incongruent feedback. For the first set of analyses, we compared the effects of congruent and incongruent feedback following the *too thin* rating from the participant, resulting in the contrast *participant rating: too thin vs. peer feedback: normal* > *participant rating: too thin vs. peer feedback: too thin*. This contrast resulted in increased activity in the left and right insula and dmPFC, extending into the ACC (dmPFC/ACC; Fig. 2a). An overview of all activated clusters for this contrast is presented in Table 1.

**Table 1:** Whole brain values for the contrast too thin–normal > too thin–too thin

Name	voxels	Brodmann area	*T*	MNI coordinates		
				*X*	*Y*	*Z*
Right inferior frontal gyrus	677	38	7.00	39	20	−14
		9	5.54	48	20	46
Right insula		47	5.76	36	20	−5
Left supplementary motor area	749	6	5.99	−9	23	67
Right superior medial gyrus		8	5.35	12	38	58
		8	5.24	15	29	61
Left insula	63	47	5.73	−30	23	−8
		38	4.05	−33	14	−17
Right parietal cortex	108	40	5.25	42	−58	55
		40	3.82	39	−52	43
		7	3.81	33	−51	58

FDR-cluster corrected at *p* < . 05, with a primary threshold of *p* < .005

For the second set of analyses, we compared the effects of congruent and incongruent feedback following the *normal* ratings from the participant. This resulted in the contrast *participant rating: normal vs. peer feedback: too thin > participant rating: normal vs. peer feedback: normal.* This contrast again showed increased activity in the left and right insula and dmPFC/ACC (Fig. 2b). An overview of all activated clusters for this contrast is presented in Table 2.

**Table 2:** Whole brain values for the contrast normal–too thin > normal–normal

Name	Voxels	Brodmann area	*T*	MNI coordinates		
				*X*	*Y*	*Z*
Right insula	522	38	7.42	36	20	−14
		47	6.69	42	20	−5
		48	5.43	30	25	10
Right middle cingulate cortex	531	32	5.46	3	29	37
Left supplementary motor area		6	4.66	−12	14	64
		6	4.57	−6	8	67
Left inferior frontal gyrus	148	38	5.01	−36	20	−17
Left insula		48	4.73	−27	23	4
Left inferior frontal gyrus		47	4.49	−33	26	−5
Right parietal/occipital cortex	325	19	4.36	30	−88	31
		37	4.25	45	−61	−8
		19	3.78	33	−91	−8
Left parietal/occipital cortex	226	19	4.28	−36	−73	−5
		37	3.94	−45	−57	−5
		19	3.48	−51	−76	−5

FDR-cluster corrected *p* < .05, with a primary threshold of *p* < .005

Hence, results showed increased activity in the dmPFC/ACC and insula in both *incongruent* situations, that is, when participants’ ratings differed from the peer feedback ratings of the media models’ body shapes.

To test whether these regions were more strongly engaged for incongruent feedback after a *too thin* rating or after a *normal* rating, these two conditions were contrasted directly to each other (*participant rating: too thin vs. peer feedback: normal* compared to *participant rating: normal vs. peer feedback: too thin*). This analysis resulted in a single cluster in the ACC that was more active for *participant rating: normal vs. peer feedback: too thin* compared to *participant rating: too thin vs. peer feedback: normal* (see Fig. 2c; Table 3). Thus, in incongruent situations participants’ ACC was more strongly activated when the peer feedback signaled that the model was *too thin* while participants rated the model as *normal*. No significant activity for the reversed contrast was shown (in the incongruent situation where *normal* peer feedback follows participants’ rating of models being *too thin*).

**Table 3:** Whole brain values for the contrast normal–too thin > too thin–normal

Name	Voxels	Brodmann Area	*T*	MNI coordinates		
				*X*	*Y*	*Z*
Right insula	152	48	5.87	30	26	7
Right inferior frontal gyrus		48	4.50	39	14	10
Right insula		48	3.87	39	5	7
Left precuneus	1413	5	5.54	−9	−49	61
Left precentral gyrus		6	5.41	−33	−10	46
Left superior temporal gyrus	85	41	4.91	−51	−31	16
		48	4.28	−57	−31	22
		21	3.57	−63	−28	7

FDR-cluster corrected at *p* < . 05, with a primary threshold of *p* < .005

#### Relation with self-esteem

**Fig. 3:**
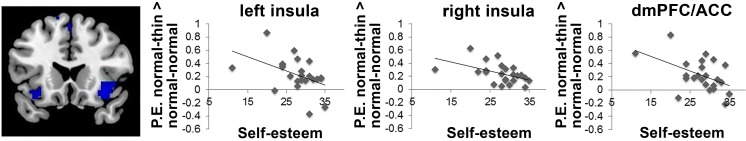
ROI plots of left insula, right insula, dmPFC/ACC, and self-esteem (Color figure online)

To test for relations between neural activity to peer feedback and self-esteem, ROIs from the left and right insula and dmPFC/ACC were extracted from the general contrast incongruent–congruent feedback across conditions (see Method sections). For these three regions, contrast values were computed for *participant rating: too thin vs. peer feedback: normal* minus *participant rating: normal vs. peer feedback: normal* and for *participant rating: normal vs. peer feedback: too thin* minus *participant rating: too thin vs. peer feedback: too thin*. Significant negative correlations were found between self-esteem and activity in both left insula (*r* = −.45, *p =* .03), right insula (*r* = −.49, *p =.*02) and the dmPFC/ACC (*r* = −.48, *p =* .02), but only for the contrast *participant rating: normal vs. peer feedback: too thin* (vs, *participant rating: normal vs. peer feedback: normal*). These relations were not found for the other incongruent contrast (*participant rating: too thin vs. peer feedback: normal* compared to *participant rating: too thin vs. peer feedback: too thin*, all *p*s > .17). Thus, neural activity associated with receiving feedback that peers considered the model too thin whereas their own rating was *normal*, was stronger for those with lower self-esteem (illustrated in Fig. 3).

Finally, to test whether other constructs related to body image were also related to neural activation in the contrast *participant rating: normal vs. peer feedback: too thin* (vs. *participant rating: normal vs. peer feedback: normal*), the same analyses were also performed with BMI and body dissatisfaction. Neither BMI nor body dissatisfaction was correlated with any of the contrast values. For an overview of all correlations, see Table 4.

**Table 4:** Display of correlations between the dmPFC/ACC, left insula and right insula ROIs, and self-esteem, BMI and body dissatisfaction

ROI	dmPFC/ACC NT-NN	L Insula NT-NN	R Insula NT-NN	dmPFC/ACC TN-TT	L Insula TN-TT	R Insula TN-TT
Self-esteem	*r* = −.48*	*r* = −.45*	*r* = −.49*	*r* = −29	*r* = .06	*r* = .07
BMI	*r* = −.01	*r* = −.07	*r* = −.24	*r* = .05	*r* = −.04	*r* = −.10
Body dissatisfaction	*r* = .31	*r* = .10	*r* = .13	*r* = .01	*r* = .10	*r* = .06

Significant correlations (*p* < .05) are indicated with an asterisk (*)

NT = normal–too thin; NN = normal–normal; TN = too thin–normal; TT = too thin–too thin

## Experiment 2

### Method

#### Participants

The sample for the behavioral study consisted of 34 healthy female participants ages 18 to 21 years (*M*_age_ = 19.2, *SD*_age_ = 1.0). Participants were recruited through local advertisements and through a recruitment website. All participants’ height and weight were measured to calculate their BMI (based on weight in kilos divided by the square height in meters, cf. WHO, 2015). This resulted in an average BMI of 21.9 (*SD* = 1.9; range: 17.4–27.2). Participants received €10 for participation. This study was approved by the University’s Medical Ethical Committee and was conducted in accordance with the provisions of the World Medical Association Declaration of Helsinki.

#### Task

The Body Image Paradigm was exactly similar to the paradigm used in Experiment 1, but no jitter was included between the trials. After completing the experiment the participants filled out two filler questionnaires that were not related to the experiment. Subsequently, they performed the Body Image Paradigm again, this time without peer feedback. The outcome measures were participant ratings (*normal weight* vs. *too thin*) of the same pre-categorized *normal weight* and *too thin* models in Session 2 (without peer feedback) versus Session 1 (with peer feedback). After completion of the task, they completed the self-esteem, body dissatisfaction and weight/height questionnaires.

### Results

#### Behavioral results

As in Experiment 1, we tested participants’ ratings of media models as being *too thin* or *normal* compared to our initial precategorized models as being *too thin* or *normal* (i.e., *precategorized: too thin vs. participants’ rating: too thin*; *precategorized: too thin vs. participants’ rating: normal*; *precategorized: normal vs. participants’ rating: too thin*; *precategorized: normal vs. participants’ rating: normal*). In Session 1 (with peer feedback), 67.0% of the media models that were precategorized as *too thin* were also rated as *too thin* in the main study, while 94.4% of the media models that were precategorized as normal models were rated as *normal weight*.

Next, we tested whether these ratings changed in the subsequent assessments (Session 2) when all the models were rated again (without peer feedback). If participants changed their assessments in the direction of the feedback from Session 1, it would be expected that precategorized *too thin* models would be more often rated as *normal*, and that precategorized *normal* models would be more often rated as *too thin*, given that the peer feedback provided 50% incongruent feedback. A 2 (model type) × 2 (session) repeated-measures ANOVA was conducted where model type distinguished between models that were precategorized as *too thin* or *normal*, and session distinguished between ratings in Session 1 (with feedback) and Session 2 (without feedback). The ANOVA resulted in a main effect of model type, *F*(1, 33) = 24.58, *p* < .001, and a significant Model Type × Session interaction, *F*(1, 33) = 4.67, *p* < .05. A post hoc test revealed that for precategorized *too thin* models there was no significant change in ratings from Session 1 (*M* = 67.0, *SD* = 24.1) to Session 2 (*M* = 69.5, *SD* = 25.2), *F*(1, 33) = 2.49, *p* = .12. In contrast, for precategorized *normal* models, a decrease was found in *normal* ratings from Session 1 (*M* = 94.4, *SD* = 6.6) to Session 2 (*M* = 92.1, *SD* = 9.2) *F*(1, 33) = 4.48, *p* < .05. These results show that only for precategorized *normal* models, the peer feedback influenced participant ratings in the subsequent assessment, such that participants more often rated these models as *too thin* in Session 2.

#### Body-related measurements

Interrelations between BMI, self-esteem, and body dissatisfaction were again examined using correlation analyses. There were no significant outliers. A significant negative correlation was found between self-esteem and body dissatisfaction (*r* = −.44, *p* < .05), indicating that a lower self-esteem relates to experiencing more body dissatisfaction. A significant positive correlation was found between BMI and body dissatisfaction (*r* = .54, *p* < .01), indicating that a higher BMI relates to experiencing more body dissatisfaction. No significant correlation was found between BMI and self-esteem (*r* = −.15, *p* = .45).

Changes in ratings from Session 1 to Session 2 were not significantly correlated with BMI, body dissatisfaction, or self-esteem.

## General discussion

In the current media-based society, the vast portrayals of too-thin body ideals pose a significant threat to healthy body perceptions and self-esteem in (late) adolescent girls. However, there is little research on how these media portrayals interact with opinions of peers, even though it is well-known that during adolescence, peers are highly relevant to adolescents, and being socially accepted is an important part of social development (Sebastian, Viding, Williams, & Blakemore, 2010). Therefore, the goal of this study was to elucidate underlying mechanisms in responding to peer feedback on late adolescent girls’ ratings of media model images in terms of *normal* or *too thin* by using neural measures. Specifically, the peer feedback could be consistent with or deviate from girls’ own ratings of bikini models’ body sizes.

We first tested how participants rated the media model images, which were preselected on perceived body weight while controlling for attractiveness. The ratings of the media models’ body sizes as *normal* or *too thin* generally converged with our pretest results, indicating that the selection of media models was successful in our study samples. Interestingly, regardless of their own ratings of media models’ body sizes (i.e., rating the model as *too thin* or *normal*), it took participants longer to rate the precategorized *too thin* models compared to the precategorized *normal* models. Such a finding implies that evaluating the body size of thin-ideal model images required more deliberation time than evaluating the body size of normal model images. From a cultivation perspective (the theory that one’s world view is heavily influenced by one’s exposure to social media; Gerbner, Gross, Morgan, Signorielli, & Shanahan, 2002), it can be assumed that the overrepresentation of thin-ideal bodies in contemporary media has led to accepting thin-ideal bodies as normal among females (cf. Veldhuis et al., 2014a). Although cultural differences should be acknowledged, similar cultivation processes among non-Western minority children are found (Veldhuis, te Poel, Pepping, Konijn, & Spekman, 2017). Possibly, it is more important for late adolescent girls to give the commonly available thin-ideal bodies the *correct* rating (i.e. rate *thin* models as normal, because media implies those body sizes are normal). Therefore they may have required longer deliberation for the *too thin* precategorized models (independent of whether the adolescent girl’s rating was *too thin* or *normal*). Being confronted with the choice may have instigated some reflection on whether the thin-ideal actually is normal.

To address the question what potential mechanisms underlie the influence of peer feedback on body perceptions at a neural level, we examined how late adolescent girls’ own ratings versus peer feedback on the models’ body sizes affected neural activity in congruent and incongruent conditions. The results showed increased activity in the dmPFC/ACC and insula in incongruent situations: that is, when the participant rated the model as *normal* while the peer feedback indicated the model as *too thin*, or vice versa. The observed increased activity corresponds to previous findings that these brain regions become active in case of deviant peer feedback (Berns et al., 2010; Klucharev et al., 2009) and in uncertain situations, such as when social norms are violated (for a review, see Rilling & Sanfey, 2011). Interestingly, this effect was stronger in the ACC when participant ratings of a media model as being of *normal* body size were followed by peer feedback indicating that the media model is *too thin*. This result suggests stronger effects in case of peer feedback deviating from the late adolescent girls’ norm of the thin-body ideal. Perhaps, bodies that were rated as *normal* by the participants were not expected to be rated as *too thin* by peers in the incongruent condition, precisely because they were quite certain about these ratings (given the precategorization results; 91.7% overall for the *normal* models). In coalescence with a cultivation perspective on media’s impact, the peer feedback then has a larger impact in correcting one’s view into the direction of what is assumed to be normal. This underlines our theoretical notion of the combined influence of media and peers in setting a standard for what is considered to be normal (i.e., *too thin*).

The finding of stronger ACC activation for incongruent feedback to a *normal* rating also fit with the results of Experiment 2, which showed that participants actually changed their behavior in the direction of the feedback. This result is consistent with prior research in adults showing that peer feedback has a conformity effect in subsequent ratings (Klucharev et al., 2009; Zaki et al., 2011). In line with the neural results from Experiment 1, the change in behavior was only found for models that were precategorized as *normal* (and after the feedback rated more often as *too thin*). Possibly, especially perceptions of normal weight models are influenced by the opinion of the peer group, such that the peer group may influence the opinion of the participant towards model ratings. Alternatively, ratings of the precategorized *normal* models were more consistent with precategorization (91.7%), allowing more possibilities for the influence of peer feedback. Future research should test this directionality effect in more detail.

Finally, in Experiment 1 we found that especially late adolescent girls with lower self-esteem were impacted more by incongruent feedback from peers when directed toward what should be considered normal body shapes, as indicated by associations between neural activity in dmPFC/ACC and bilateral insula, and self-esteem. This effect was independent of their own BMI and mirrors previous findings that girls with lower self-esteem were more affected by peer feedback on ultrathin images compared to those higher in self-esteem (Veldhuis et al., 2014a). We found no association between self-esteem and change in behavior after receiving per feedback. These findings suggest that some effects can be visible in neural activity in the absence of behavioral differences, possibly because the observed behavioral change ratings were subtle.

Our findings have several implications for the understanding of late adolescents’ body ideals, which seem to be biased towards unhealthily thin body ideals. The finding that neural responses to deviance between one’s own rating and peer feedback is especially the case for girls with lower self-esteem, suggests that these girls are possibly more prone to peer influence. In future studies, it will be important to examine how self-esteem and peer feedback are related to healthy body images in later stages of life (Neumark-Sztainer, Paxton, Hannan, Haines, & Story, 2006).

The current study also had some limitations that should be addressed in future research. First, the study was limited to girls in the age range 18–21 years. Future studies should examine sensitive periods in adolescent development by also including younger adolescents. Second, different participant samples (though similar in many respects) were included in the fMRI study and in the behavioral feedback conformity study. Therefore, it was not possible to link the neural responses directly to behavioral change. Finally, the feedback presented was from unknown and ostensible peers; therefore, it remains an important question how late adolescent girls respond to feedback from real-life friends or known peers. As our results from Experiment 2 indicated, it is possible to change ratings of media models from *normal* to *too thin* after receiving peer feedback. Another interesting direction for future research will be to examine if peers may also have a *positive* effect on body image, specifically in girls with lower self-esteem. For example, to further enhance late adolescent girls’ body image, peer feedback and comments can be used in interventions by providing clearer normative indications of what can be considered *normal* and healthy body sizes, and present the variations in body sizes that generally exist.

Future research should also test systematic variations in feedback content (e.g., descriptive, comparative, evaluative, and motivational feedback; see Hawkins, Kreuter, Resnicow, Fishbein, & Dijkstra, 2008) and in body sizes of the portrayed media models. Such an approach will provide a solid background to define which peer feedback mechanisms are used best in media-based interventions to influence participants on what media imagery is normal, realistic, and healthy. Importantly, moderating factors regarding individual susceptibility should be included to explain who is more susceptible to the media-by-peer interactions than others (cf. Ferguson, 2013; Veldhuis et al., 2014a). Also, relatively novel media settings, such as Facebook, Instagram, and YouTube, where images and direct feedback from others on such images go hand in hand, provide valuable and relevant environments to manipulate and test such media-by-peer feedback interactions (Konijn, Veldhuis, & Plaisier, 2013). In all, our results indicate that media-based peer feedback as an intervention strategy may hold great promise for future research and applications.

To conclude, this study uniquely combined neuroimaging and behavioral measures to further investigate the mechanisms and effects of media imagery perceptions. Subsequently, the results hold implications for using peer feedback in (media-based) interventions in order to set the young women open to discussion of which body images and sizes are healthy and normal, and which are not.
